# Minimal Residual Disease in the Management of B-Cell Acute Lymphoblastic Leukemia: A Systematic Review of Studies from Indian Settings

**DOI:** 10.1007/s12288-023-01641-6

**Published:** 2023-03-30

**Authors:** Hari Menon, Pawan Kumar Singh, Bhausaheb Bagal, Tuphan Dolai, Ankita Jain, Antara Chaudhri

**Affiliations:** 1 Hematology and Head Medical Oncology, St John’s National Academy of Health Sciences, Bangalore, Karnataka India; 2 Hemato Oncology and Bone Marrow Transplant, BLK-Max Centre for Bone Marrow Transplant, Delhi, India; 3 Medical Oncology, Tata Memorial Hospital, Mumbai, Maharashtra India; 4 Hematology Department, NRS Medical College and Hospital, Kolkata, West Bengal India; 5 Oncology and Field Medical, Pfizer Oncology, Mumbai, Maharashtra India; 6 Pfizer Oncology, Mumbai, Maharashtra India

**Keywords:** Minimal residual disease, Acute lymphoblastic leukemia, Multiparametric flow cytometry, Real-time quantitative PCR, End-of-induction, India

## Abstract

Minimal residual disease (MRD) has become an essential tool in the management of B-cell acute lymphoblastic leukemia (B-ALL) and aids in tailoring treatment strategies to suit specific patient needs. Although much progress has been made in this area, there is limited data on the use of MRD in the Indian context. Our objective was to identify relevant literature that discusses the utility of MRD in the management of B-cell ALL in adolescents and young adults (AYA) and adults in Indian settings. A systematic search and screening of articles were performed using the Preferred Reporting Items for Systematic Reviews and Meta-Analysis (PRISMA) guidelines. The primary data source was PubMed followed by Google Scholar for articles and conference proceedings. Of the 254 records screened, 24 records were retained for analysis. MRD monitoring had a significant role in the management of AYA/adult B-cell ALL patients. Variability of results was observed across these studies with respect to methods, techniques, and use. However, these studies evidenced and validated the importance of MRD assessment in risk-adapted management of B-cell ALL and highlighted the need for optimization. The advances in MRD diagnostics and applications are yet to be tested and adopted in Indian settings. Hence, there is a need for in-depth research to develop and optimize approaches for calibrating country-specific management strategies. The potential role of MRD assessments in anticipating relapse or treatment failures warrants more attention for the preemptive positioning of novel strategies involving immunotherapies.

## Introduction

Acute lymphoblastic leukemia (ALL) has a varied clinical presentation, with precursor B-cell ALL (B-ALL) being one of its most common immunological subtypes and affecting almost 75% of adult ALL cases. The most frequently observed genetic aberration in B-cell ALL is the Philadelphia chromosome-positive ALL (Ph + ve ALL) [1, 2]. The American Cancer Society estimates the occurrence of 6600 new cases of ALL in their population in the year 2022 [3], whereas the incidence rate of ALL in India has been estimated to be 101.4 per million and 62.3 per million, across all age groups and genders, respectively [2]. Minimal residual disease (MRD) has emerged as a robust prognostic indicator in B-cell ALL [4]. MRD can be described as the presence of a very low number of cancerous/malignant cells after chemotherapy or following a hematopoietic stem cell transplantation (HSCT) [5, 6].

MRD detection in ALL dates to the 1980s when immunofluorescence microscopy was used [7]. The use of MRD diagnostics in clinical trials as a surrogate endpoint for evaluating the efficacy of novel agents has also been observed. It was initially used for T-cell ALL because a highly specific immunophenotype for B-cell ALL had not been identified [8, 9]. The limitation of two- or three-color immunofluorescence microscopy made the detection of minor differences in marker expressions challenging, hence many new techniques emerged [8, 9]. Polymerase chain reaction (PCR), flow cytometry (FCM), next-generation sequencing (NGS), and next-generation flow cytometry are the molecular techniques used currently for MRD assessment [10, 11].

MRD assessments are very time-point specific [10]; hence, they are a critical prognostic indicator in very high-risk B-cell ALL patients. Among these patients, the subset that fails to achieve the end-of-induction (EOI) MRD positivity displays inferior outcomes [12, 13]. In addition, patients displaying very early MRD clearance have significantly better outcomes [14]. Studies have shown a direct strong correlation between MRD and the risk of relapse in ALL patients, thereby highlighting the prognostic value of MRD [10, 15]. Results from a meta-analysis reflect that pretransplant MRD positivity is a significant negative predictor of relapse-free survival (RFS), event-free survival (EFS), and overall survival (OS) [16]. Such results emphasize the importance of MRD evaluation before transplant, especially when treatment intensification is needed.

There is a paucity of data on MRD from an Indian context, and a complete understanding of MRD assessments and their correlation with outcomes among B-cell ALL patients is also lacking [17]. The primary objective of this review was to survey the available literature that discusses different aspects of MRD testing in the Indian context, specifically focusing on adolescent and young adults (AYA) and adult B-cell ALL patients. The insights gathered from studies specific to Indian settings are presented in this systematic literature review and discussed considering the global research landscape.

## Methods

### Literature Search

A systematic literature search was done using the Preferred Reporting Items for Systematic Reviews and Meta-analyses (PRISMA) reporting guidelines [18]. After the finalization of objectives, specific research questions were framed to guide the search process. Search queries were designed and reviewed independently for coverage and accuracy. The review of queries was based on the Peer Review for Electronic Search Strategies (PRESS) Guidelines. The components of the search string used in combinations are listed in Table 1.

**Table 1:** Components of the query included as a part of the search strategy

S. no.	Filter	Query string
1	Age filter	((Adult) OR (Adolescent*) OR (AYA) OR (Old) OR (Elderly))
2	B-ALL	((“Precursor Cell Lymphoblastic Leukemia-Lymphoma”[mh]) OR (“Precursor B-Cell Lymphoblastic Leukemia-Lymphoma”[mh]) OR (“B-ALL”) OR (“B-cell ALL”) OR ((b cell) AND (acute lymph* leukemia)))
3	MRD	("Neoplasm, Residual"[mh] OR "MRD" OR "Measurable residual disease" OR "Minimal residual disease" OR "Minimal/Measurable residual disease")
4	R/R	((relapse*) OR (refractory))
5	Prognosis or risk factors	((prognosis) OR (risk*))
6	Treatment outcomes	(“Treatment Outcome”[mh] OR “Survival” OR “Disease-Free Survival”[mh] OR “Progression-Free Survival”[mh] OR "Complete Remission")
7	Additional filters	((India) NOT ((case report) OR (news) OR (consensus) OR (review)) AND (y_10[Filter]))

### Data Sources

The primary source of literature was PubMed. Additional searches were performed on Google Scholar, and the first 200 results were considered for preliminary screening [19]. From this subset, only articles not indexed in PubMed were included. Articles published within the last ten years from the date of query execution (April 3, 2022) were selected. Other data sources where the search was extended included original abstracts presented at the annual meetings of (1) the American Society of Hematology (ASH); (2) the American Society of Clinical Oncology (ASCO); (3) the European Society for Medical Oncology (ESMO); (4) the Indian Society of Hematology and Blood Transfusion (ISHBT); and the European Hematology Association (EHA) during the period 2019–2021.

### Study Screening and Data Extraction

Based on objectives and research questions, the inclusion/exclusion criteria were outlined. The titles and abstracts of the articles retrieved were screened. Two independent reviewers performed the screening process, and disagreements were resolved by agreement based on discussion. Full-text versions of the articles that met the inclusion criteria were retrieved. These articles were selected for the next round of screening based on the full-text review. Articles that met the screening criteria were further considered for the extraction of study-relevant data. A predefined structured template was used for capturing data.

### Screening Criteria

To minimize the risk of bias, all screening and evaluation steps were carried out independently by two individuals. Final decisions were made after resolving the disagreements based on discussion among reviewers. In addition, the inclusion/exclusion criteria were predefined based on which a template was designed to collect data. The risk of bias assessment of studies using standardized checklists (e.g., Newcastle–Ottawa scale) was not carried out as the primary goal was to survey the MRD-specific methodological aspects and application areas, and the number of articles retrieved was limited.

## Results

### Summary of Search

**Fig. 1:**
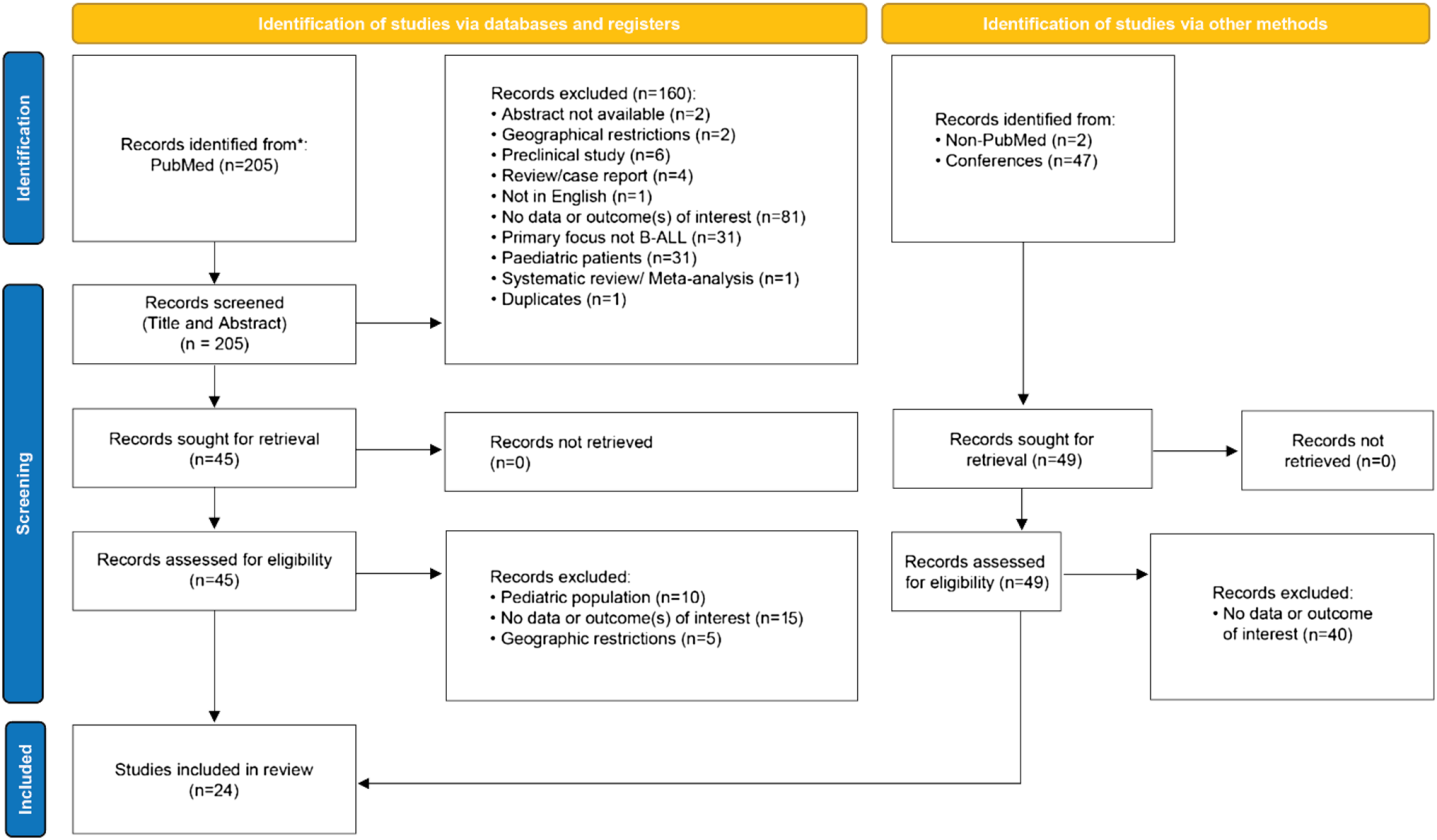
The process and flow diagram for screening and identifying study-relevant literature. Framework adapted from the Preferred Reporting Items for Systematic reviews and Meta-Analysis guidelines. B-ALL: B-cell acute lymphoblastic leukemia

We identified and screened 205 studies from PubMed. An additional two articles were identified by searching Google Scholar for articles not indexed in PubMed. A total of 47 studies (range 2019–2021) were retrieved from annual meetings of ASH, ASCO, EHA, ESMO, and ISHBT. Of the total 254 studies, 24 articles were shortlisted according to the inclusion/exclusion criteria and after the removal of redundant articles by two independent reviewers. The detailed search strategy adopted during the systematic review is shown in Fig. 1. The list of final articles included after screening is given in Table 2.

**Table 2:** List of articles that met the final inclusion criteria

References	No. of patients	Study objective	Study-relevant findings
*Journal articles*			
Bommannan et al*.* [20]	14	Difficulties faced during diagnosis and MRD assessment of de novo CD19-negative and dim B-cell ALL patients	Identifying robust alternatives to CD19 will help in better diagnosis and follow-up
Ganesan et al*.* [21]	1383	Outcomes in AYA ALL patients	Patients treated with pediatric and adult protocols had no difference in their induction outcomes concerning the achievement of CR, induction mortality, or MRD positivity rate
Bommannan et al*.* [22]	152	Clinical–pathological profiling of CD56- and CD7-expressing B-cell ALL patients	Patients with high-risk disease and EOI MRD positivity were at higher risk of adverse events
Das et al*.* [23]	239	Evaluating the expression of CD123 in acute leukemia, comparing it with post-induction morphologic complete remission and MRD status	CD123 may be considered as a cardinal marker for: Residual disease assessment Response evaluation in AML and B-cell ALL
Jain et al*.* [24]	35	Testing the activity of bortezomib and rituximab with a pediatric-inspired regimen during induction therapy in newly diagnosed adolescents and adults with CD201, Ph-negative precursor B-cell ALL	The combination of bortezomib, rituximab, and a pediatric-inspired ALL regime was well tolerated in the following cases: End-of-induction MRD-negative status was achieved in 70.9% of patients MRD-negative rates improved to 87.5% after consolidation Event-free survival and overall survival rates were 78.8% and 78.7%, respectively
Jain et al*.* [25]	507	Evaluating if intensifying therapy for high-risk patients yielded improved results	Intensified therapy in the high-risk subset is associated with a significant increase in early treatment-related mortality and cost of treatment A modified GMALL regimen was cost-effective
Virk et al*.* [26]	478	Prospective study of TSLPR expression in 478 consecutive B-cell ALL patients and its correlation with various hematologic parameters and EOI MRD	TSLPR-positive patients did not show a significantly higher MRD compared to TSLPR-negative cases (37% vs. 33%)
Pandey et al*.* [27]	130	Evaluation of outcomes (post-induction response rates, MRD, and OS) with modified MCP 841 in pediatric and AYA ALL	MRD-negative patients did better than those with MRD-positive status, 29 vs. 22 months (p = 0.03)
Rajendra et al*.* [28]	349	Outcomes and prognostic factors in the treatment of AYA ALL with a pediatric-inspired regimen	MRD persistence after induction emerged as the only factor predictive of poor outcomes
Arunachalam et al*.* [29]	94	Evaluating the prognostic relevance of MRD based on *BCR-ABL1* copy numbers in Ph-positive ALL patients	Molecular MRD based on *BCR-ABL1* copy number ratio is an ideal prognostic indicator in Ph-positive ALL patients undergoing treatment
Garg et al*.* [30]	75	Analyzing frequency of CD34 expression in B-cell ALL in Indian patients and determining its prognostic significance	CD34 expression does not associate with known prognostic markers in B-cell ALL CD34 negativity was not associated with adverse prognosis concerning MRD or cytogenetics
Patkar et al*.* [31]	10	Flow cytometry-based MRD assay for BCP-ALL with emphasis on assay standardization and cost	A cost-effective MRD panel is applicable to over 90% of patients
Panda et al*.* [17]	104	Flow cytometry-based MRD assay for ALL with emphasis on the determination of the number of patients who had MRD on day 35 of induction therapy and its correlation with the outcome and other prognostic factors	MRD correlates with certain known prognostic factors Though EFS and OS were lower in MRD-positive patients, the results were not statistically significant, probably because of the small sample size
Chatterjee et al*.* [32]	214	Expression of CD304/neuropilin-1 in adult B-cell ALL patients and evaluation of its practical utility in MFC-based MRD analysis	CD304 is commonly expressed in adult B-cell ALL It distinguishes B-cell ALL blasts from normal precursor B cells A stable MRD marker is distinctly useful in the detection of MFC-based MRD monitoring
Das et al*.* [33]	281	MRD assessment by MFC, using a combination of Difference from Normal (DFN) and LAIP approach and used of baseline immunophenotype (IPT) for MRD assessment	A single 10-color panel tube with LAIP and DFN approach was found to be a reliable tool for MRD assessment(diagnosis and time points) CD 45,CD19, CD34, CD10, CD20 and CD38 was one panel and CD123, CD81, CD58 and HLA-DR was another panel CD58 was the most frequent LAIPs observed at diagnostic and MRD time points in over 85% of the cases CD123 was found to be relevant in detecting LAIP at both time points in over 50% of the cases Changes in at least one of the nine immunophenotypic markers in B-ALL post- induction was observed in 94.04% cases
*Conference abstracts*			
Aboobacker et al*.* [34]	191	Role of allo-SCT in the management of both newly diagnosed and relapsed patients with ALL	Allo-SCT is an effective option for high-risk diseases in CR1 and late relapses Limited benefit in patients with active/refractory disease and those with early relapse of disease
Lakshmy et al*.* [35]	37	Feasibility of using a low-cost, low-intensive regimen of bortezomib + vincristine + prednisolone for salvage therapy after relapse of ALL	The use of bortezomib-based salvage chemotherapy resulted in 35% remission rates in patients with relapsed B-cell ALL with minimal toxicity Can be easily administered on an outpatient basis
Ozcan et al*.*[36]	22	Evaluating the efficacy and safety of two dose levels of inotuzumab in adults with R/R ALL, eligible for HCT and have a higher risk of post-HCT SOS	A starting dose of 1.2 mg/m^2^/cycle inotuzumab showed acceptable efficacy, with half of the patients achieving remission, and > 70% of those in remission being MRD negative
Bhandary et al*.* [37]	35	Expression of LAIP markers at diagnosis in BCP-ALL for optimization of MRD panel	CD9, CD81, CD73, and CD86 were the most relevant markers, which can be included in a single tube MRD panel (CD45, CD19, CD20, CD10, CD34, CD38, CD9, CD81, CD73, and CD86) Cost-efficient and reduces the number of LAIP markers currently used for MRD diagnosis
Meganathan et al*.* [38]	184	Evaluating the use of a 12-color flow cytometry panel for the diagnosis of acute leukemia with a sequential strategy	A 12-color panel is: Cost-effective Provides more information, which helps in the diagnosis of rare/atypical cases and follow-up MRD assessment
Vatsala et al.[39]	54	Assessing the diagnostic role of flow cytometry in immunophenotyping of adult ALL	MFC-based immunophenotyping enhances the traditional morphological diagnosis Also aids in monitoring the disease during MRD assessment
Mazumder et al*.* [40]	82	Proposing an optimized 10-color panel for MRD detection based on the LAIP expression at diagnosis	A single 10-color tube comprising CD45, CD19, CD34, CD10, CD20, CD38, CD73, CD86, CD81, and CD44/CD58 for diagnosis as well as for MRD in the post-therapy samples of BCP-ALL
Dhar et al*.* [41]	29	Evaluating the significance of expression of CD38, CD58, CD49d, and CD66c in ALL	Incorporating prognostic markers such as CD38, CD58, CD49d, and CD66c at the time of diagnosis: Helps provide valuable information on disease progression Aids MRD analysis at a later stage for disease and therapy-response monitoring
Arunachalam et al*.* [42]	403	The clinical significance of EOI MRD monitoring in B-cell ALL	The 4-tube, 4-color panel has wider applicability than the 2-tube, 8-color panel It demonstrated a positive MRD in a higher percentage of patients The survival worsened for every log increase in the MRD value

*allo-SCT* Allogeneic stem cell transplantation, *ALL* Acute lymphoblastic leukemia, *AML* Acute myeloid leukemia, *AYA* Adolescent and young adults, *BCP-ALL* B-cell precursor ALL, *BCR-ABL1* Breakpoint cluster region-Abelson murine leukemia 1, *BiTE* Bispecific T-cell engagers, *CAR-T* Chimeric antigen receptor T-cell, *CD* Cluster of differentiation, *CR* Complete remission, *EFS* Event-free survival, *EOI* End of induction, *GMALL* German multicenter ALL, *HCT* Hematopoietic cell transplantation, *SOS* Sinusoidal Obstructive Syndrome, *LAIP* Leukemia-associated aberrant immunophenotype, *DFN* Difference from Normal, *IPT* Immunophenotype, *HLA-DR* Human Leukocyte Antigen-DR istotype, *MFC* Multiparametric flow cytometry, *MRD* Minimal residual disease, *OS* Overall survival, *Ph* Philadelphia, *R/R* Relapsed/refractory, *TSLPR* Thymic stromal lymphopoietin receptor

### MRD Assessment: Current Status

The Hematology Cancer Consortium maintains a database (Indian Acute Leukemia Research Database) that stores retrospective data from nine centers across India. Our search identified only one study exploring this database. The data published by Ganesan et al*.* [21] reflected that MRD assessment was available for only 47% of patients in the AYA group. Of the 1383 patients registered, 1141 (82.5%) underwent treatment, and MRD status was available for 654 patients. After induction, 76% of patients achieved complete remission (CR), and MRD was positive in 240 of 654 (37%) patients. Both univariate and multivariate analyses highlighted that inferior EFS and OS were associated with MRD positivity [21].

### Timing of MRD Assessment

Regarding the timings for assessment, five studies followed EOI assessments on days 29–33 [22–26], except for studies by Chatterjee et al*.* [32] and Das et al*.* [33] that made assessments between days 35 and 40 and 30 and 35, respectively [27]. Individual studies (n = 2) that evaluated at mid-induction on day 21 or after phase 1a induction were also identified [27, 28]. Only three studies included end-of-consolidation (EOC) evaluations and subsequent follow-ups [24, 29, 32].

### Samples and MRD Detection Methods

A majority of studies (n = 10) included in this systematic review used bone marrow aspirate, with a few (n = 3) using peripheral blood as well. Data comparing outcomes between these two samples were not available. Of the 24 studies, 22 studies used FCM using multicolored panels and multiparametric flow cytometry (MFC) for MRD assessments, whereas two used real-time quantitative PCR (RQ-PCR) [25, 29]. MFC methods with 5-color [30], 8-color [27, 42], 10-color [23, 24, 26, 28, 32, 33, 40], and 12-color [38] panels were observed in 12 studies. The panel of markers identified in these studies includes CD10, CD13, CD19, CD20, CD23, CD34, CD66c, CD123, CD200, and CD304 for the FCM analysis, and RQ-PCR was used for the *BCR-ABL1* transcript characterization. A study by Chatterjee et al*.*[32] evaluated the expression pattern of CD304 in a cohort of adult B-cell ALL patients and reported that CD304 was found to be positive in a significant percentage of EOI (62/129 [48%]) and EOC (26/50 [52%]) MRD-positive, B-cell ALL samples. CD123 has also received consideration as a marker for residual disease assessment and response evaluation in acute myeloid leukemia and B-ALL. CD123 expression at diagnosis was shown to be associated with post-induction MRD-positive status in B-ALL (*p* < 0.001) [23, 33]. The Leukemia-associated aberrant immunophenotype (LAIP) and Difference from Normal (DFN) approach was found to be a reliable tool for MRD assessment at the diagnostic and MRD time points in the study by Das et al*.* [33]. Changes in at least one of the nine immunophenotypic markers in B-ALL post-induction was observed in 94.04% cases. The utility of other markers such as CD44, CD73, CD304, and CD200 was assessed in those cases requiring review of baseline IPT. Out of 18 cases, the information was useful in 8 cases (44.4%). The study emphasized the need for better markers for distinguishing leukemic blasts from hematogones. Additionally, MRD assessment in B-ALL is complicated by changes in IPT after induction chemotherapy, necessitating pattern recognition and simultaneous analysis of multiple IPT markers. [33]. Arunachalam et al*.* [29] analyzed the prognostic relevance of MRD based on *BCR-ABL1* copy numbers in Ph-positive ALL patients. *BCR-ABL1* copy numbers were evaluated using RQ-PCR. The cost-effectiveness of the MRD method was analyzed in three studies [25, 31, 38]. Patkar et al*.* [31] proposed a relatively cost-effective MRD panel applicable to over 90% of patients. Using their approach, they detected MRD in 60% and 47% of patients at mid- and end-induction time points, respectively. Another two studies focused on the optimization of MRD panels with the goal of cost-effectiveness and reduction in the number of LAIPs [22, 37].

### MRD and Treatment Outcomes

Only five articles focused on evaluating specific treatment regimens with MRD as a measure of post-induction response [24, 27, 28, 35, 36]. The outcomes of patients treated with different regimens were presented as combined results in most studies. Only one study was identified, where post-induction MRD was used as one of the indications for stem cell transplantation (SCT) in the first complete remission (CR1) [34]. A phase 2 study evaluating the combination of bortezomib, rituximab, and a pediatric-inspired ALL regimen showed post-induction persistent MRD to be associated with inferior OS and EFS [24]. Outcomes among MRD-based, risk-stratified patients show that patients with poor-risk status were associated with inferior OS and EFS [29]. The impact of MRD on OS was studied in pediatric and AYA groups treated with a modified multicenter protocol (MCP) 841 [27]. The results outline that MRD-negative patients responded better than MRD-positive patients (*p* = 0.03). Post-induction MRD was acknowledged as a useful prognostic tool for ALL patients treated with the modified MCP 841 protocol. Furthermore, in a study assessing the outcomes associated with Berlin–Frankfurt–Münster-90 among AYA, post-induction MRD persistence emerged as the only factor predictive of poor outcomes [28].

### Risk Category and MRD Status

A study conducted by Arunachalam et al*.* [29] assessed the prognostic relevance of MRD based on *BCR-ABL1* copy numbers in Ph-positive ALL patients. In this study, the MRD status was assessed at three different time points. Patients having persistent MRD-positive status at all three measured time points or having an increasing *BCR-ABL1/ABL1* copy number ratio with an increase in their MRD-positive status by the third measurement were categorized as MRD poor-risk status. Those patients having MRD negativity and decreasing *BCR-ABL1/ABL1* copy number ratio with a strong MRD-negative status by the third assessment were categorized as MRD good risk. MRD poor-risk patients had adverse outcomes when compared to MRD good-risk patients in terms of OS (*p* = 0.031) and EFS (*p* ≤ 0.001). Patients with high-risk diseases and those with EOI MRD positivity are at higher risk of adverse events [22]. CD304 positivity has also been shown to be associated with *BCR-ABL1* fusion, with a significant percentage of EOI and EOC MRD positivity [32]. Results from a smaller cohort indicate that patients with P2Y receptor family member 8-cytokine receptor-like factor 2 (P2RY8-CRLF2) translocation who underwent EOI MRD testing showed positivity [26].

## Discussion

The primary objective of this study was to survey the available literature that discusses different aspects of MRD testing in the Indian context, specifically focusing on AYA and adult B-cell ALL patients, as B-cell ALL studies done so far have been limited to the pediatric population and have been done on smaller cohorts. A systematic search was carried out, which indicated limited data on the use and impact of MRD assessment among AYA and adult B-cell ALL patients in Indian settings. Available data on samples, timings, techniques, and outcomes were gathered. This review presents and discusses the limitations and aspects that need further investigation. The MRD assessment status was available only from one study [21].

The aspects of sensitivity of MRD assessment and its implications have not been discussed in the identified studies. Samples, sensitivity, and timings can be critical factors in leveraging maximum benefits from MRD assessment. An important point to consider is the timing of MRD measurement, which can help in taking treatment decisions. Different insights can be provided by MRD, depending upon the timing of assessment: very early, after induction/consolidation, and before and after SCT. Although early response assessment (days 8 and 15) has been discussed in the literature, the review of articles selected for this study did not reveal any discussion on such aspects. Evidence supports early MRD testing [1]. Negative MRD status at very early time points during the induction phase correlates with better outcomes both in adult and in childhood B-ALL [43, 44]. Our results also suggest that MRD was a critical prognostic indicator strongly associated with RFS and EFS [29]. The focus of most studies identified in the review was on assessment at EOI and/or EOC. The importance of EOI MRD assessment was highlighted in one study [42]. Different treatment protocols have established informative checkpoints, which aid in monitoring outcomes appropriately. However, such sequential monitoring was not observed in most studies identified in Indian settings.

The majority of studies identified from Indian settings have used bone marrow samples, with some studies using peripheral blood. However, no comparison of utility has been studied. Several clinical studies have evaluated MRD status in bone marrow samples and blood in B-cell ALL and T-cell ALL [33, 45–47]. The use of peripheral blood MRD can serve as a noninvasive technique to monitor systemic relapse and might have additional clinical and diagnostic value in patients with a high risk of extramedullary disease [48]. There is a paucity of data on studies evaluating the application of MRD assessment. Typically, MRD assessments are done using a single aspirate sample, which can vary due to sampling error and/or collection techniques [49]. Inaccuracies resulting from a sample that is diluted or has an unequal distribution of disease involvement in the bone marrow can pose limitations.

The use of > 10^−4^ or > 5 × 10^−4^ as a threshold has been suggested for poor MRD responders with poor prognoses [4]. The acceptable level of sensitivity of MRD assays remains unresolved [1]. MFC and quantitative PCR are the most frequently used MRD detection techniques/methods in clinical practice. MRD-based risk stratification can be further refined by using NGS like sensitive assays. Accurate identification of patients with persistent MRD who are at the highest risk of relapse will allow the design of reasonable post-remission therapies using novel agents [50]. Our results suggested that CD304 was a stable MRD marker that could be useful in detecting MFC-based MRD monitoring, especially in high-sensitivity MRD assay [32]. Even though LAIP and DFN approach combination is one of the best for MRD assessment, its utility could be affected by the immunophenotypic patterns of leukemia blasts mimicking hematogones and in CD10 dim to negative cases. Hence, every case with a hematogone pattern and dim to negative CD10 expression at diagnosis is recommended to have a statement in the diagnostic flow cytometry report so that the hemato-pathologist viewing the report is aware of this. More immunophenotypic markers should be evaluated which can help in differentiating between hematogones and leukemia blasts, thereby improving the reliability of the MFC-based MRD assays. Changes in immunophenotypic markers in B-ALL post- induction are frequent and may be useful but such changes could possibly compromise the MRD assessment in certain cases [33].

MRD assessment for monitoring treatment outcomes was one of the objectives defined for this review. The Programa Español de Tratamientos en Hematología (PETHEMA) ALL-AR03 trial used MRD to guide treatment decisions at the EOC and found that HSCT could be avoided in patients who reached MRD negativity without adversely affecting their prognosis [51]. MRD can play a role in sparing patients from risks associated with transplantation without negatively affecting survival outcomes [52]. Patients who are at a high risk of leukemia relapse after allogeneic SCT can be identified by the kinetics of MRD clearance. Patients who have not been able to achieve early molecular remission after transplantation might require prompt and appropriate preventive treatments [53]. The role of MRD in the management of Ph-positive B-cell ALL has also been established and can be important in in-patient stratification [14]. The percentage of MRD reduction corresponds with superior disease-free survival (DFS), irrespective of the tyrosine kinase inhibitor (TKI) used [54]. MRD persistence and/or reappearance can be indicative of resistant mutations (e.g., T315I). Such cases may warrant alternative approaches, including novel TKIs and/or combinations of TKI with immunotherapy [55]. The early achievement of MRD negativity in the treatment of adults with Ph-negative B-cell ALL is a strong predictor of survival [56]. Ph-negative patients in this study were classified as B-cell ALL patients who had achieved MRD-negative status at the end of induction at two different time points and were also observed to be early MRD responders. MRD also has the potential to guide the selection of patients for treatment de-intensification. However, the appropriate way to utilize MRD results for treatment de-intensification is yet to be defined.

The value of having achieved MRD negativity is significant in pediatric and adult ALL patients [15]. In adult B-ALL patients, achieving MRD negativity is consistently associated with better survival outcomes than those of patients with MRD-positive status [1]. Such results have been consistent across methods, therapies, times of MRD assessment, cutoff levels, and disease subtypes [1]. Relatively few reports are available on the significance of MRD in patients with relapsed disease. Such status was also evident from the search carried out for this study. In adults with ALL, the prognostic significance of MRD in relapsed/refractory ALL has been primarily reported in individual studies using novel salvage treatments [57]. In a study of inotuzumab as salvage therapy, achieving MRD negativity was associated with a longer remission duration [58]. A retrospective analysis of 78 patients showed a differential impact of MRD negativity according to salvage (S) status in patients with relapsed/refractory B-ALL [57]. Patients with relapsed/refractory ALL who achieved MRD negativity in S1 had long-term survival, whereas patients in S2 generally had poor outcomes regardless of MRD status. Patients in S1 who achieved MRD negativity and subsequently underwent SCT had the best outcomes, with a 2-year OS rate of 65%. Assessment of the prognostic value of MRD negativity at the end of inotuzumab treatment shows that patients in first salvage who achieved MRD negativity experienced significantly improved survival vs. that seen in MRD-positive patients. This observation was significant, particularly among those patients who proceeded to SCT. Among patients with relapsed/refractory ALL treated with inotuzumab, the MRD-negative complete remission/complete remission with incomplete count recovery (CR/CRi) group had the best survival outcomes [59]. The benefit of achieving MRD negativity highlights its relevance for assessing prognosis and measuring treatment efficacy.

Although studies identified in the search reveal minimal data on applications of MRD, there is a broader research landscape with extended scope of utility. MRD response has been considered in drug development as an early marker of efficacy in clinical studies. It has potential use as a surrogate endpoint in the registration of studies for accelerated drug approval [60, 61]. MRD status warrants consideration as an early measure of disease response for evaluating new therapies, improving the efficiency of clinical trials, accelerating drug development, and regulatory approval [15]. However, approval of such findings based on an intermediate endpoint would require confirmation using traditional efficacy endpoints.

## Conclusion

The systematic search carried out as a part of this study revealed limited data on applications of MRD in the management of B-cell ALL among AYA and adult populations. The existing data suggest its applicability in facilitating improved treatment outcomes. The comparison of results from included studies with the scope of published evidence from literature databases highlights the need for more research specific to Indian settings. Aspects related to cost, resource limitations, and differences in biology have been pointed out. These may be important considerations in designing future research investigations. Current evidence suggests that MRD is an essential tool to facilitate the optimal course of management of B-cell ALL by assisting in critical clinical decisions. Such assessments can effectuate the distinctness of situations where the use of conventional options has higher chances of treatment failure and identify patients who can benefit the most from novel agents.
